# Feasibility of Present-Centered Therapy for Prolonged Grief Disorder: Results of a Pilot Study

**DOI:** 10.3389/fpsyt.2021.534664

**Published:** 2021-04-15

**Authors:** Anna Vogel, Hannah Comtesse, Agnes Nocon, Anette Kersting, Winfried Rief, Regina Steil, Rita Rosner

**Affiliations:** ^1^Department of Psychology, Catholic University Eichstaett-Ingolstadt, Eichstaett, Germany; ^2^Faculty of Applied Healthcare Science, Deggendorf Institute of Technology, Deggendorf, Germany; ^3^Department of Psychosomatic Medicine and Psychotherapy, University of Leipzig, Leipzig, Germany; ^4^Department of Clinical Psychology and Psychotherapy, Philipps-University of Marburg, Marburg, Germany; ^5^Department of Clinical Psychology and Psychotherapy, Institute of Psychology, Goethe University, Frankfurt, Germany

**Keywords:** prolonged grief disorder, loss, psychotherapy, bereavement, present-centered therapy

## Abstract

Present-centered therapy (PCT) was originally developed as a strong comparator for the non-specific effects of psychotherapy in the treatment of posttraumatic stress disorder. PCT qualifies as a not strictly supportive treatment as it is structured and homework is assigned between sessions. It does not focus on cognitive restructuring or exposure. A growing body of literature supports its beneficial effects. For example, it demonstrated only slightly inferior effect sizes and lower dropout rates compared to that of trauma-focused cognitive behavioral therapy in several trials with patients suffering from posttraumatic stress disorder. The current study is the first to evaluate the feasibility and the treatment effects of PCT in adults with prolonged grief disorder (PGD). Meta-analyses on psychotherapy for PGD have yielded moderate effect sizes. *N* = 20 individuals suffering from PGD were treated with PCT by novice therapists as part of a preparation phase for an upcoming RCT in an outpatient setting. Treatment consisted of 20–24 sessions á 50 min. All outcomes were assessed before treatment, at post-treatment, and at the 3-month follow-up. The primary outcome, PGD symptom severity, was assessed using the Interview for Prolonged Grief-13. Secondary outcomes were self-reported PGD severity, depression, general psychological distress, and somatic symptom severity. Furthermore, therapists evaluated their experiences with their first PCT patient and the treatment manual. In intent-to-treat analyses of all patients we found a significant decrease in interview-based PGD symptom severity at post-treatment (*d* = 1.26). Decreases were maintained up to the 3-month follow-up assessment (*d* = 1.25). There were also significant decreases in self-reported PGD symptoms, depression, and general psychological distress. No changes were observed for somatic symptoms. The completion rate was 85%. Therapists deemed PCT to be a learnable treatment program that can be adapted to the patient's individual needs. The preliminary results of PCT as a treatment for PGD demonstrate large effects and indicate good feasibility in outpatient settings. The treatment effects were larger than those reported in meta-analyses. Thus, PCT is a promising treatment for PGD. Possible future research directions are discussed.

## Introduction

Prolonged grief disorder (PGD) has emerged as a well-defined mental disorder, distinguishable from major depression and posttraumatic stress disorder (PTSD) or other stress-related disorders (1). It has now been included in the ICD-11 (2), with slightly different criteria to those of its counterpart in the DSM-5, the “persistent complex bereavement disorder” (3). Earlier concepts of PGD encompassed complicated or traumatic grief [e.g., (4, 5)]. The core symptoms of all concepts are intense yearning and preoccupation with the deceased; reactive distress symptoms, such as feeling stunned or shocked by the loss; avoidance of reminders of the reality of the loss and emotional numbing, and finally social/identity disruption, for instance feeling detached or finding it difficult to trust others (6). The symptoms and impairment have to persist for more than 6 months after the death of a significant other.

PGD rates have varied considerably across studies due to methodological heterogeneity, sample demographic features, and loss-related characteristics. While a recent meta-analysis has found that 1 in 10 bereaved adults following non-violent death of a loved one suffer from PGD (7), representative studies report lower prevalences [e.g., 7% conditional prevalence in (8)]. Higher prevalences are associated with unnatural losses, with nearly half of the bereaved persons experiencing PGD (9). PGD has been found to be associated with both psychological and physical morbidity, such as impaired quality-of-life (10), increased risk of comorbid disorders with high rates of depression, PTSD, and anxiety disorders (11), increased suicidality (12) and deteriorated health (13). The negative consequences of PGD indicate a need for efficacious treatments. Still, there are relatively few controlled studies examining psychological treatments for PGD. Boelen and Smid (14) list three recommended psychological therapies that have been tested in at least two independent and controlled studies, including “complicated grief treatment” [including elements of exposure, cognitive restructuring, and interpersonal therapy; (15–17)], cognitive behavioral therapy [CBT; combining exposure and cognitive interventions; (18, 19)], and internet-based CBT [encompassing exposure, cognitive interventions, and behavioral activation applied using writing assignments; (20, 21)].

Meta-analyses on the treatment of complicated, traumatic or prolonged grief yielded effect sizes (ESs) for grief outcomes between 0.53 for those with clinically relevant symptoms (22), 0.53 for those undergoing psychotherapy (23), and 0.45 overall, and 0.58, when only considering those treated who were minimally 6 months post-loss (24). The respective ESs for depressive symptoms ranged between 0.16 (22) and 0.35 (24). ESs for general mental distress were reported as small [*d* = 0.26 in (24)]. In recent reviews (14, 24) on PGD treatment research, it was found that most interventions were grief-specific approaches and used exposure, cognitive restructuring, behavioral activation, and elements of interpersonal therapy. The authors also state that there are too few randomized controlled trials (RCTs) with active controls and that dismantling studies are missing. Consequently, no conclusions can be drawn about the active ingredients of successful treatment manuals.

Present-Centered Therapy [PCT; (25)] was originally developed as a strong comparator for the non-specific effects of psychotherapy in the treatment of PTSD. The goals of PCT are to enhance interpersonal connectedness, improve patients' insight into their current symptoms, and promote a greater sense of mastery via use of effective approaches to solving problems. Therefore, it involves empathic listening and support from the therapist as well as the basic components of behavioral therapy, namely education about the links between symptoms and daily problems, and the fostering of problem-solving skills, including homework exercises. The treatment is provided within the context of intentionally compassionate and helpful acts of the therapist based on client-centered principles like offering empathy, unconditional positive regard and congruence and is led by the individual daily stressors and problems the patient presents. PCT excludes specific trauma-focused components (i.e., exposure, cognitive restructuring of dysfunctional beliefs, stress inoculation training), and therefore seems to choose an alternative approach to address avoidance: by actively dealing with the current problems that may have arisen as a result of the traumatic event, but without engaging emotionally with the traumatic event itself.

In an older meta-analysis based on five RCTs, PCT showed good results in PTSD treatment and lower dropout rates than trauma-focused approaches (26). In a recent Cochrane review (27) based on 12 RCTs, PCT was found to be superior to waitlist (SMD = −0.84). A comparison of PCT to trauma-focused CBT did not support PCT non-inferiority. ESs differed for PCT and trauma-focused CBT with 0.32 in favor of trauma-focused CBT. PCT resulted in 16% lower dropout rates than trauma-focused CBT. Current treatment guidelines suggest that PCT may be offered as a treatment for PTSD when trauma-focused CBT is either not available or not preferred by the patient (28).

Taken together, PCT can be deemed to be what is known as a bona fide therapy, namely a therapy based on psychological principles containing specific factors (i.e., specific techniques like fostering of problem-solving skills and homework exercises, or promoting a theory of the therapeutic change, namely by client-centered principles of offering empathy, unconditional positive regard and congruence) and delivered by trained professionals [cf. (29, 30)]. It is, therefore, a credible intervention for both patients and therapists. Unlike almost all other interventions that have been examined in patients suffering from PGD so far, PCT does not include exposure or cognitive interventions. Given that patients with PGD, as well as those with PTSD, suffer from avoidance (albeit to slightly different degrees), an intervention that takes a different approach to addressing avoidance seems to represent an interesting alternative to previously studied interventions. Furthermore, on a theoretical basis, by focusing on the active mastery of daily problems and functional coping, one might speculate that PCT resembles restoration-orientation according to the Dual Process Model of coping with bereavement (31). This is why we decided to adapt PCT to the needs of patients suffering from PGD. If PCT for PGD would prove feasible and clinical impactful, it might not only serve as an active bona fide treatment with a different treatment focus most PGD interventions had so far, but it also promises to be an ideal active control condition in future PGD trials. Therefore, the aim of this study was to evaluate the feasibility and the treatment effects of PCT in adults with PGD, and to explore therapists' and supervisor's experiences with this new treatment.

## Methods

### Participants

Participants were treatment-seeking adults aged 18 to 75, whose losses had occurred at least 6 months previously. A primary diagnosis of PGD, as assessed in the Interview for Prolonged Grief-13 [PG-13; (6, 32), see below], was required for inclusion. Because of the ongoing discussion about a multiplicity of different criteria sets for PGD (2, 3, 6, 33, 34), and because the final ICD-11 criteria were not available when we started this trial in 2017, we decided on a compromise between criteria according to Prigerson et al. (6) and the not yet finalized ICD-11 (2). To meet the criteria for PGD in the current study, it was necessary for participants to report at least (a) one separation distress symptom (rated as ≥4 on a 5-point-scale: 1 = never/not at all, 5 = several times a day/extremely), (b) four out of nine cognitive, emotional, and behavioral symptoms (each symptom rated as ≥ 4), and (c) significant impairment in social, occupational or other important domains, for 6 months or longer, after the loss according to the PG-13, see below. Patients had to have sufficient cognitive and German language skills, and give their written informed consent. If patients were on antidepressant medication, the treatment regime needed to be stable for at least 4 weeks prior to joining the trial. The exclusion criteria were: (1) current psychotic or severe substance use disorder, or acute suicidality; (2) ongoing psychotherapy; (3) participation in another treatment trial; and (4) continuous treatment with benzodiazepines, antipsychotics, or opioids. Any change in psychotropic medication during the course of the study was continuously monitored.

### Procedure

The current trial was an integral part of a preparation phase for an RCT [(35), German Clinical Trials Register, ID: DRKS00012317]. Treatment was offered at four University outpatient mental health clinics in Germany. The study was approved by the Institutional Review Board of the Catholic University Eichstaett-Ingolstadt (2016/21), and by three Institutional Review Boards of the other study centers (Ethics Committee of the Department of Psychology and Sports of the Goethe University Frankfurt, Ethical Committee at the Medical Faculty of Leipzig University, Local Ethics Committee of the Department of Psychology of the University of Marburg). Recruitment efforts included a study website, advertisements in public and social media, newspaper and radio interviews, flyers in family practices, health and community centers, or churches, and informing general and mental health practitioners via mailings, as well as via talks and publications in the specialized press. The first patient started therapy in June 2017, the last patient finished therapy in May 2019.

The trial included assessments at baseline, post-treatment, and at the 3-month follow-up, each comprising the same clinical interviews and self-ratings described below. All assessments were conducted by trained clinical raters who were blind to the participants' baseline assessment results and treatment progression. Study safety was ensured by monitoring for the incidence of serious adverse events (e.g., suicide attempts, death, occurrence of life-threatening conditions, events that lead to physical disability) using a therapist/rater-administered checklist every treatment session and at post-treatment and follow-up. Participants received a small financial compensation for taking part in the post-treatment and follow-up assessments (€20 for each assessment).

Treatment was administered by 20 study therapists, who were predominantly female (94%), master's level psychologists in advanced postgraduate clinical training (71%) and specialized in CBT (100%). All therapists were novices in PCT and were interested in taking part in an upcoming RCT. They were free in choosing training in PCT or an alternative grief-focused CBT training. All included patients represented each therapist's first training case. All PCT therapists attended a 2-day personal training course in PCT delivered by one of PCT's original authors, Dr. Shea. During the trial, therapists were supervised bi-weekly at the respective study center. In addition, they participated in centralized bi-weekly telephone case consultations to maintain treatment adherence. Therapeutic adherence and competence are currently being evaluated with independent ratings by two raters based on video-documented sessions selected at random.

### Outcomes and Measures

The primary outcome was the PGD severity score assessed by the PG-13 in an interview format [(6, 33), German version (36), as published in (37)], which was obtained by calculating the sum of the 11 symptom item scores (range: 11–55). Cutoff scores of 34 and 35 have been suggested for the PG-13 (37, 38). Psychometric evaluation showed good internal consistency [e.g., Cronbach's alphas from 0.83 to 0.93 in (39)]. The PG-13 was also used to assess PGD diagnostic status as defined for the current study (see above) as well as a reliable change in PGD symptoms. In the current sample, the internal consistency of the total severity score (11 items) was 0.71.

Secondary outcomes were assessed that targeted self-reported symptoms of prolonged grief, depression, somatoform symptoms, and general mental distress.

Prolonged grief symptoms were also measured using the self-report measure Inventory of Complicated Grief [ICG; (40), German version ICG-D; (41)]. Participants were asked to rate the extent to which they had experienced 19 grief symptoms during the previous month on a 5-point scale ranging from 0 = never to 4 = all the time. A prolonged grief score was computed (range: 0–76), Cronbach's alpha was 0.70. Prigerson et al. (40) suggested an ICG score >25 as the threshold for distinguishing syndromal from subsyndromal levels of PGD. However, later studies [e.g., (42, 43)] used a cutoff score of ≥ 30 as a more conservative threshold to identify clinically significant cases.

Self-reported depressive symptoms were measured using the German version of the Beck Depression Inventory II [BDI-II; (44)]. The 21 items refer to symptoms of depression during the previous 2 weeks and are rated on a 4-point scale, resulting in a total depression score ranging from 0–63. Cronbach's alpha in the current sample was 0.85.

As we had found a high level of somatoform symptoms in two earlier studies (45, 46), we decided to include a measure to specifically address somatoform complaints. The Screening for Somatoform Disorders [SOMS-7D; (47)] was used to assess self-reported somatoform symptoms. Participants were asked to rate the extent to which they had suffered from 53 somatoform symptoms during the previous 7 days on a 5-point scale (0 = not at all, 4 = very much). A somatization severity index was calculated ranging from 0–208. Cronbach's alpha was 0.89 in the current sample.

Self-reported general mental distress was measured using the Global Severity Index (GSI) from the German version of the Brief Symptom Inventory [BSI; (48)]. The BSI is a widely used 53-item measure of subjective distress caused by psychological and somatic symptoms over the previous seven days. Responses are scored on a 5-point scale (0 = not at all, 4 = extremely). The GSI is calculated using the sums for the nine subscales plus the four additional items, and divided by the total number of items to which the individual responded (score range: 0–4). In the current sample, Cronbach's alpha of the BSI-GSI was 0.94.

To obtain information about the study therapists' evaluations of PCT, we asked all study therapists to fill in an online questionnaire after having completed their first treatment case. We obtained ratings on four subscales: beliefs and attitudes about the intervention (i.e., individuals' attitudes toward and value placed on the intervention); design quality (i.e., perceived excellence in how the intervention is bundled, presented, and assembled); adaptability and trialability (i.e., degree to which an intervention can be adapted, tailored, refined, or reinvented to meet local needs and ability to test the intervention on a small scale in the organization); and resources and access to knowledge (i.e., level of resources dedicated for implementation and on-going operations and ease of access to digestible information and knowledge about the intervention and how to incorporate it into work tasks). For this questionnaire, we used items by Cook et al. (49) or generated items based on the framework model of Damschroder et al. (50). The Consolidated Framework for Implementation Research by Damschroder et al. (50) includes the most common concepts from published implementation theories. The framework is designed to allow researchers to select the concepts most relevant for their particular setting and use them to evaluate the implementation process. As we were primarily concerned with implementing a new intervention, PCT, within the well-established structures of four University outpatient clinics, we selected concepts relating to the characteristics of the intervention, individual characteristics and the inner setting of our clinics to evaluate the implementation process of PCT. Therefore, for the purpose of this study, we created four subscales to evaluate the implementation process of PCT from the viewpoint of therapists. Overall, the questionnaire consisted of 23 items to be rated on a 5-point scale (1 = I do not agree at all, 5 = I agree fully). Mean scores were calculated for each of the four subscales (mean score range: 1–5). One item on barriers to the implementation of PCT also included the option of giving an answer in an open format, if applicable. In the current sample, the internal consistencies of the beliefs and attitudes about the intervention scale (9 items; α = 0.78), the design quality scale (3 items; α = 0.80), and the resources and access to knowledge scale (5 items; α = 0.78) were good. The internal consistency of the adaptability and trialability scale (4 items; α = 0.70) was acceptable. See Supplementary Material 1 for the questionnaire.

The presence of comorbid mental disorders according to DSM-IV criteria at baseline was determined using the Structured Clinical Interview for DSM-IV Axis I [(51), German version: (52)]. DSM-IV criteria were used, because no validated German version for the Structured Clinical Interview for DSM-5 was available in 2017 yet. Interviewer-rated acute suicidality was assessed using the Columbia-Suicide Severity Rating Scale [C-SSRS; (53)]. The five items in the intensity of suicide ideation subscale were used to rate the intensity of current suicide ideation on 5- and 6-point scales (score range: 2–25).

### Intervention

The PCT manual for PTSD (25) was adapted in cooperation with Dr. Shea for its use with PGD patients (54). To adapt PCT for treating PGD in Germany, we made the following modifications: (1). In order to make PCT a credible intervention for both patients and therapists, session length and number were adapted to the standard health insurance coverage for outpatient CBT treatments in Germany, that is, 20 50-min sessions (as compared to 10 100-min sessions). (2). Educating the patient at the beginning of treatment focused on grief-specific topics including grief symptoms and their relation to problems in day-to-day life. Furthermore, therapists were allowed to collect information relevant to the loss in the first sessions in order to establish a therapeutic relationship. (3). Up to four additional optional sessions were possible to handle special occasions or needs (e.g., suicidality, dealing with anniversaries). Altogether, a maximum of 24 therapy sessions was possible according to the protocol, see Table 1 for an overview. Besides educating the patients on grief symptoms, PCT did not include any grief-specific cognitive-behavioral components (e.g., exposure, cognitive restructuring of dysfunctional beliefs, instructions to do specific homework). Its focus was on the daily monitoring of stressors and problems in their relation to PGD and on their active mastery. The therapists provided support and established an empathic relationship. This encouraged the expression of thoughts or feelings and explicitly focused on factors of client-centered therapy. Therefore, PCT has elements of supportive therapy, but is a more structured approach that follows a manual and includes the use of a diary to record problems throughout the week. It did not, however, use any active interventions except for giving information, pointing out themes, or other ways of fostering functional coping and the patient's problem-solving skills. If the patient brought up problems regarding the loss, discussions on the loss itself were avoided in favor of focusing on how to better cope with symptoms in daily life resulting from the loss. The aim was to achieve greater insight and support regarding the consequences of the loss. When patients were emotionally distressed during sessions, therapists acted in an empathic, compassionate and helpful manner and promoted functional problem-solving when appropriate.

**Table 1:** Treatment protocol of PCT adapted for PGD.

**Session number**	**Treatment strategies**
1–2	Present an overview of the program, discuss the general rationale for the treatment approach (aims of the study, session frequency, etc.), answer client's questions and identify areas of concern, collect information relevant to the loss using a PGD interview guideline. Homework: filling-in relevant forms.
2–3	Ask about the reaction to the first session, present agenda for the current session, educate the patient about frequent reactions to loss. Homework: reading the handout on frequent reactions to loss.
3–4	Present the agenda for the current session, clarify the background and the methods of the treatment, explain the use of daily monitoring in the diary card (recording of events or problems). Homework: filling-in the diary card, reading the handout on the treatment.
4–17	Revise the diary card, establish the agenda for the current session, problem-solving focused on difficulties identified by the patient. Homework: filling-in the diary-card.
18	Follow format for session 4, prepare the patient for impending termination.
19	Follow format for session 4.
20	Review the past week, review the progress in treatment, terminate therapy saying goodbye.

*PCT, present-centered therapy; PGD, prolonged grief disorder*.

### Data Analyses

All primary and secondary outcome analyses were performed as intent-to-treat (ITT) analyses. We used the last observation carried forward (LOCF) procedure to replace missing values due to participants dropping out of the study. To examine the effects of PCT on primary and secondary outcomes, we conducted a repeated-measures multivariate analysis of variance (MANOVA) with measurement time points (pretreatment, post-treatment, follow-up) as a within-subject factor. To assess the effects on each of the outcome measures, repeated measures analyses of variance (ANOVAs) were performed with three measurement time points *post hoc*. The significance level for all analyses was set to α = 0.05 (2-tailed). As analyses were considered in an exploratory manner, the significance level was not adjusted for multiple tests. Cohen's *d* ES was calculated for within group pre-post comparisons. Cochran's *Q* test, which is a generalization of the McNemar test for more than two measurement time points, was applied to investigate the change in diagnostic status with respect to PGD (55). Statistics were calculated using IBM SPSS Statistics 25 for Windows. The criterion for a clinically reliable change in the PG-13 severity score according to Jacobson and Truax (56) was calculated on the basis of Cronbach's α of the PG-13 severity score in the current study, as proposed by Martinovich et al. (57). Thus, clinically reliable improvement was defined as a reduction of more than 6.88 points in the PG-13 severity score.

The open format answers regarding barriers to implementation of the PCT survey were analyzed and summarized by creating condensed meaning units according to Bengtsson (58).

## Results

### Participant Flow

**Figure 1:**
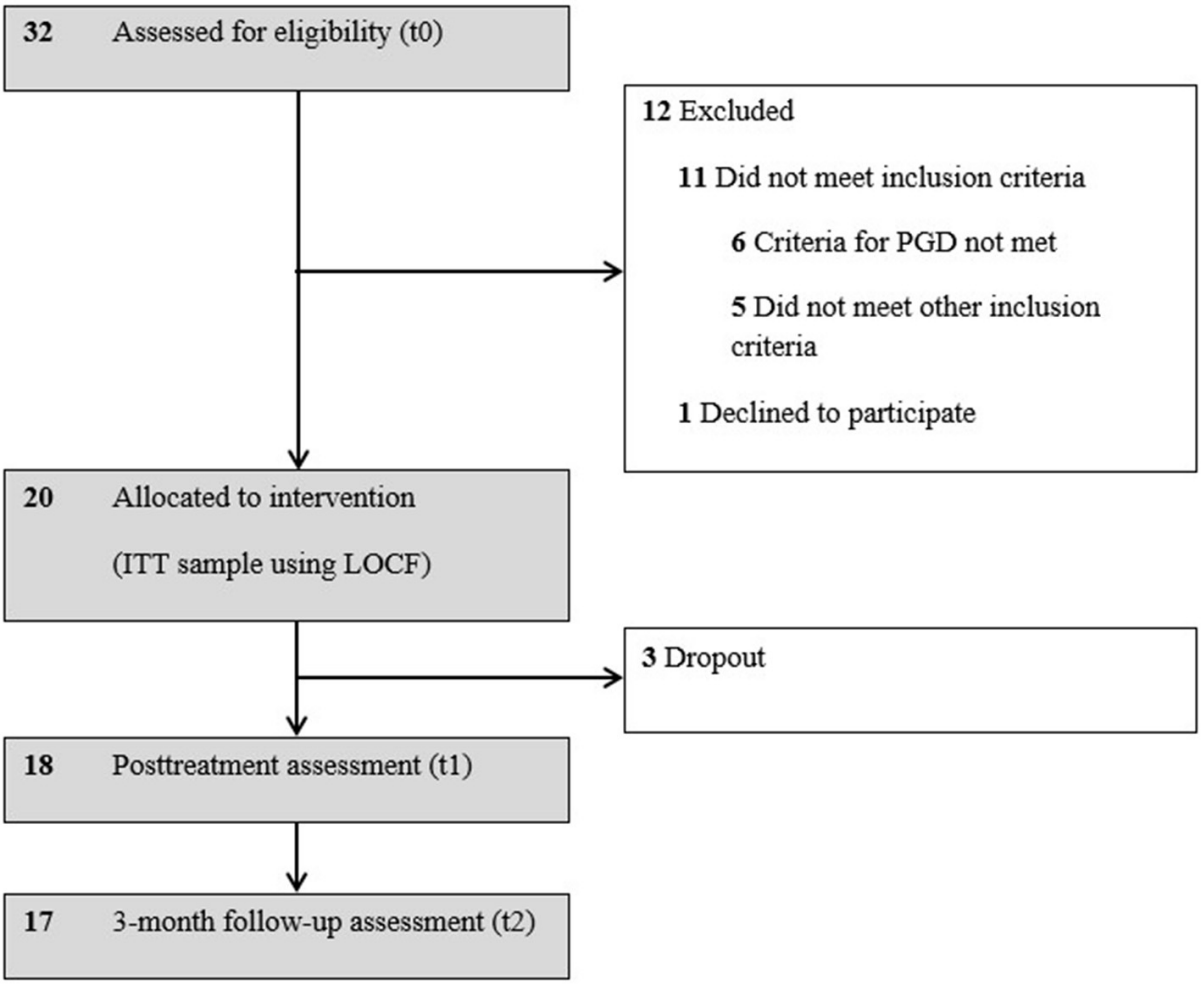
Flow diagram of study participants. ITT, intent-to-treat; LOCF, last observation carried forward; PGD, prolonged grief disorder.

We screened 32 individuals for eligibility; 12 of them did not meet the study criteria. Six of them had not been clinically diagnosed as having PGD. See Figure 1 for participant flow. One eligible candidate for treatment declined. As a result, 20 participants began treatment with PCT. Their ages ranged from 37 to 74, with *n* = 3 aged under 50.

Most participants were women (80%) and currently employed (50%). Fifteen participants had completed secondary education only (≤ 12 years), and five had been to college (>12 years). The majority of participants had lost a partner (45%), or child (35%). The deaths were mostly natural (75%) but unexpected (50%). The mean time since loss was 48.8 months (*Mdn* = 27.0; range: 6–337). All patients met the criteria for PGD diagnostic status as defined for the current study.

At baseline, 16 participants (80%) met the criteria of at least one comorbid psychiatric disorder with an average of *M* = 1.00 (*SD* = 0.65) in addition to PGD. For further details, see Table 2.

**Table 2:** Sociodemographic and loss-related characteristics of study participants in the feasibility trial of PCT adapted for PGD.

**Characteristic**	**Total sample (*N* = 20)**
**Female, % (*****n*****)**	80.0 (16)
**Age in years**, ***M*** **(*****SD*****)**	56.00 (8.84)
**Education, % (*****n*****)**
<12 years	75.0 (15)
≥12 years	25.0 (5)
**Employment, % (*****n*****)**
Employed	50.0 (10)
Unemployed	20.0 (4)
Retired	20.0 (4)
Other	10.0 (2)
**Marital status, % (*****n*****)**
Married/in a relationship	45.0 (9)
Divorced/single	15.0 (3)
Widowed	40.0 (8)
**Antidepressant medication, % (*****n*****)**	25.0 (5)
**Comorbid disorder DSM-IV^a^, % (*n*)**
None	20.0 (4)
1	60.0 (12)
2	20.0 (4)
Mood disorders	80.0 (16)
Anxiety disorders	10.0 (2)
Somatoform disorders	10.0 (2)
**Relation to the deceased, % (*****n*****)**
Child	35.0 (7)
Spouse/partner	45.0 (9)
Parent	10.0 (2)
Other	10.0 (2)
**Cause of death, % (*****n*****)**	
Natural	75.0 (15)
Unnatural	25.0 (5)
**Expectation of the death, % (*****n*****)**
Expected	40.0 (8)
Unexpected	50.0 (10)
**Time since loss in months**, ***M*** **(*****SD*****)**	48.75 (75.56)

*PCT, present-centered therapy; PGD, prolonged grief disorder*.

a *According to the Structured Clinical Interview for DSM-IV Axis I*.

Of all the participants who began treatment with PCT, three participants (15%) discontinued treatment prematurely after session 5, session 7, and session 12, respectively. In all of these cases, treatment was terminated because of a lack of treatment motivation. In two of these cases the reasons for dropout reported by the respective therapists were relatively long travel distances coupled with low motivation for change. In the third case a very low motivation to attend therapy sessions was mentioned. We were able to obtain further data from one of the participants who dropped out at post-treatment. Another participant did not complete the post-treatment self-report ratings, and data of the SOMS-7D of one further participant at the follow-up assessment was missing.

The mean duration of treatment was 23.65 weeks (*SD* = 7.10), with an average of *M* = 18.70 (*SD* = 4.99) sessions provided. Neither suicidal crises nor other serious adverse events occurred during the intervention or up to the 3-month follow-up.

### Treatment Outcome

The repeated measures MANOVA of the primary (PG-13) and secondary outcome measures (ICG, BDI-II, BSI-GSI, SOMS-7D) calculated on the basis of the ITT sample (*N* = 20), demonstrated a significant effect of time, *F*_(10, 108)_ = 2.08, *p* = 0.032.

Repeated measures ANOVA revealed a significant large effect for the total severity score of interview-rated PGD symptoms (PG-13) from pre- to posttreatment with *d* = 1.26. Improvements remained stable at the 3-month follow-up, *d* = 1.25. See Table 3 for the results of respective ANOVAs and effect sizes.

**Table 3:** Primary and secondary outcomes of study participants in the feasibility trial of PCT adapted for PGD adults based on intent-to-treat analyses with LOCF, *N* = 20.

**Outcome**	**t0**	**t1**	**t2**	**Time effect**			**t0-t1**	**t0-t2**
	***M (SD)***	***M (SD)***	***M (SD)***	***F***	***df***	***p***	***d***	***d***
PG-13	39.25 (4.61)	29.95 (9.37)	30.25 (9.08)	8.75	2	<0.001	1.26	1.25
ICG	41.70 (8.67)	29.00 (15.28)	30.70 (14.96)	5.35	2	0.007	1.02	0.90
BDI-II	26.05 (8.39)	19.75 (9.72)	18.45 (10.12)	3.71	2	0.031	0.69	0.82
BSI-GSI	1.33 (0.52)	1.02 (0.63)	0.86 (0.58)	3.48	2	0.038	0.54	0.86
SOMS-7D	30.85 (18.87)	29.20 (23.67)	24.80 (22.58)	0.41	2	0.665	0.07	0.29

*PCT, present-centered therapy; PGD, prolonged grief disorder; LOCF, last observation carried forward; t0, baseline; t1, posttreatment; t2, 3-month follow-up; PG-13, interview for prolonged grief-13; ICG, inventory of complicated grief; BDI-II, beck depression inventory; BSI-GSI, brief symptom inventory global severity index; SOMS-7D, screening for somatoform disorders*.

At post-treatment, 15 participants (75%) no longer met the PGD criteria according to the PG-13, and 13 participants (65%) at the 3-month follow-up. Three of the five cases who still met the PGD criteria after treatment, were the participants who had dropped out. The change in PGD diagnostic status over time was significant, Cochran's *Q*(2) = 24.57, *p* < 0.001. At post-treatment, 10 participants (50%) met the criteria for reliable change according to the PG-13 score, whereas 11 participants (55%) met the criteria for reliable change at the 3-month follow-up. No clinically relevant worsening of symptoms was observed. Throughout the trial, no serious adverse event was reported.

The results regarding self-reported PGD symptoms (ICG) revealed a significant large effect from pre- to post-treatment with *d* = 1.02, see Table 3. The ES remained large at the 3-month follow-up, *d* = 0.90. However, total sum scores at posttreatment as well as 3-month follow up remained at or above more conservative cutoff scores used in other studies [e.g., (43)].

Participants improved significantly from pre- to post-treatment and up to the 3-month follow-up with regard to BDI-II and BSI-GSI. ESs for improvements of depressive symptoms and general mental distress were medium to large, ranging from 0.54 to 0.86. There were no significant improvements with regard to somatoform symptoms as assessed by the SOMS-7D.

### Evaluation of the Study Therapists' Perspective

Of all the PCT study therapists, 17 (85%) responded to our online questionnaire after having completed their first treatment case, including the three therapists whose patients discontinued treatment prematurely. Their demographic characteristics are given in Table 4.

**Table 4:** Demographic characteristic of study therapists in the feasibility trial of PCT adapted for PGD.

**Characteristic**	**Total sample (*N* = 17)**
**Female, % (*****n*****)**	94.1 (16)
**Age in years**, ***M*** **(*****SD*****)**	32.41 (5.85)
**Education, % (*****n*****)**	
Licensed psychotherapist	29.4 (5)
Master's level psychologist in advanced postgraduate clinical training	70.6 (12)
**Specialization in cognitive behavioral therapy, % (*****n*****)**	100 (17)
**Duration of clinical work in months**, ***M*** **(*****SD*****)**	50.88 (44.76)
**Number of treated cases**, ***M*** **(*****SD*****)**	26.75 (51.03)

*PCT, present-centered therapy; PGD, prolonged grief disorder*.

The mean scores of the four subscales of the evaluation of PCT can be considered as medium to high, with *M* = 3.65 (*SD* = 0.54) regarding beliefs and attitudes about the intervention, *M* = 3.94 (*SD* = 0.64) regarding design quality, *M* = 3.94 (*SD* = 0.64) regarding adaptability and trialability, and *M* = 3.95 (*SD* = 0.74) regarding resources and access to knowledge. Therefore, PCT adapted for PGD as evaluated by the study therapists seems to be an adaptable and learnable intervention of good design quality that was evaluated positively by CBT therapists.

Only nine therapists (53%) reported at least one barrier to administering PCT with their pilot cases. The reported barriers were grouped into five categories. In three cases (17.6%), therapists reported that features of a personality disorder or rigid behavior patterns complicated implementing the manual with their patient. Three categories were each mentioned by two therapists (11.8%) as a barrier: patient's lack of motivation for change or reactance, lack of permission to use typical CBT methods (like cognitive restructuring, exposition), and problems during treatment because the patient would have needed clearly defined goals. One therapist (5.9%) reported the PCT manual as being not specific enough as a further problem when administering PCT. Two of these categories (personality features and rigid behavior patterns, reactance from the patient and lack of motivation for change) seem to be associated with the individual patient's characteristics and might not, therefore, be a problem specific to PCT. The other three categories (lack of target definition with the patient, unspecific manual, no permission to use typical CBT methods) seem to constitute specific problems associated with differences between the nature of the PCT program and the prior CBT-training of the therapists.

### Experiences With PCT From the Supervisor's Perspective

Multicenter case consultations were put in place to monitor treatment adherence throughout the study. Study therapists became easily engaged in PCT, reported generally positive relationships with their patients and no important problems with regard to treatment adherence. They also reported that most patients engaged very well in PCT. Some therapists expressed doubts about the effectiveness of the PCT treatment and were reminded of the body of evidence that had been published up to then. All therapists had a CBT qualification, including mastery of a minimal amount of client-centered techniques. The group repeatedly discussed appropriate and inappropriate therapist behaviors to stay within the limits of the treatment manual. Another challenging question was how important problem solving and filling in the diary card were. Some therapists, for example, reported, that patients did not fill in the diary cards or carry out specific homework assignments. During case consultations therapists were motivated to focus more on the therapeutic relationship as mode of action rather than following a CBT-typical perspective when dealing with homework assignments undone. In most cases a decision was taken to focus on behaviors that enhance a positive relationship and promote factors of client-centered therapy. With respect to problem-solving, therapists were reminded to first help the patient to identify their emotions, and only thereafter to work on problem-solving, thus avoiding the risk of talking about solutions that were trivial and not specific enough.

## Discussion

### Outcomes and Safety of PCT for PGD

This study is the first to investigate the feasibility of PCT in patients suffering from PGD. While PCT previously had been evaluated in samples with PTSD (27), we were interested in the feasibility and treatment effects of PCT in a sample of bereaved adults. We found significant pre- to post-treatment reductions in PGD symptoms as assessed by the PG-13 and the ICG indices with large ESs. However, PCT did not yield ICG total sum scores substantially below clinical cutoffs at post-treatment or follow-up. According to the PG-13 severity score, 50% of participants showed clinically meaningful improvement, and 75% of the participants achieved remission from PGD. Improvements remained nearly stable at the 3-month follow-up. Regarding secondary outcome measures, significant improvements from pre- to post-treatment were observed with respect to depressive symptoms and general mental distress as assessed in self-report, with medium pre-post ESs and large ESs at follow-up.

The ESs regarding PGD symptoms were higher than those reported in meta-analyses for PGD treatments in adults (22–24). These results are encouraging as the self-reported baseline severity of PGD symptoms, *M*_ICG_ = 41.70, in our sample was comparable to several RCTs evaluating grief-focused treatments [e.g., *M*_ICG_ = 42.6 in (18), *M*_ICG_ = 47.5 in (19), *M*_ICG_ = 45.8 in (15), *M*_ICG_ = 46.1 in (59)]. Compared to small ESs for depressive symptoms and general mental distress reported in the literature (22, 24), our results with medium pre-post ESs and large ESs at follow-up seem to be promising. However, because of the small sample size and the uncontrolled design of the study, interpretation of these ESs should only be made with caution (60). Furthermore, compared to ICD-11 criteria, the more strict criteria set used in this study (6) might also have affected ESs, as including participants with lower symptom scores might have yielded in lower ESs.

The severity of somatoform symptoms at baseline was high in the current sample, as the mean score of the SOMS-7D corresponded to percentile rank = 93 according to the German norm sample (47). This is in line with results from other bereaved samples (46, 61, 62). The non-significant differences with regard to somatoform symptoms, as assessed by the SOMS-7D, might be explained by the fact that PCT does not contain any components that are assumed to be specific mechanisms of change for the treatment of somatoform disorders (63, 64).

To adapt the treatment protocol to the standard health insurance coverage for outpatient CBT treatments in Germany, we allowed 20 to 24 50-min sessions to be administered, with a mean of 18.70 sessions conducted. Therefore, the mean number of treatment sessions was considerably higher than the number reported by meta-analyses evaluating grief-focused treatments [10 in (24), 10 to 16 in (23)]. This higher number of treatment sessions might, in part, explain the large ESs we found with respect to PTG symptoms.

### Acceptability in Patients and Therapists

We did not observe any exacerbation of PGD symptoms from pretreatment to any later assessment point, nor did any adverse event occur during the trial. These results indicate that the intervention was well-received by the participants and was safe. PCT was a new treatment for all of the study therapists as PCT was previously unknown in Germany. In particular, the strong focus on client-centered techniques (following the core conditions sensu Rogers) while dispensing with almost all CBT methods, constituted an unusual approach for many of the therapists. They were all trained in CBT but had not undergone training with the focus on client-centered techniques prior to the current trial. In line with this, the lack of target definition with the patient, the unspecific manual, and the withholding of permission to use typical CBT methods seemed to be specific problems associated with the nature of the PCT program. Some study therapists reported them as barriers. However, these problems might decrease with an increasing number of cases treated with PCT and with growing expertise in emphasizing client-centered techniques in the therapeutic process. It is also questionable whether these problems occur at all with non-CBT therapists. Therefore, implementing PCT with therapists with other theoretical backgrounds than CBT in future studies would be very informative.

Three participants dropped out of treatment (15%). In two of these cases, the respective patients had to travel very long distances to the treatment site combined with a reported low motivation for change, as indicated by their therapists. In the third case very low motivation to attend therapy sessions was mentioned from the start. All dropouts may also be associated with the participants' high pretreatment PG-13 severity (all above the median of 39) and ICG scores (all above the median of 40). However, in terms of completion rates, the results in our study are good, with 85% completion in PCT vs. 82% in the study by Shear et al. (59), 73% in the study by Shear et al. (15), 79% in the study by Rosner et al. (46) and 71% in the study by Boelen et al. (10).

Overall, the promising results of PCT in patients suffering from PGD as studied in this trial, but also in previous trials with PTSD patients, raise questions about possible psychological mechanisms that are responsible for change in PCT. Unlike many effective interventions for PTSD and PGD, PCT clearly does not include any exposure or cognitive restructuring. It can be assumed that exposure and cognitive restructuring cause direct symptom reductions in patients via facilitating emotional processing, minimizing avoidance, and modifying negative cognitions—mechanisms that seem to be crucial for recovering from PTSD (65) as well as PGD (66). In contrast, patients treated with PCT seem to experience enhanced psychosocial functioning through the application and practice of more effective solutions to daily stressors, and this might indirectly lead to a symptom reduction. PCT's focus on problem-solving in the present might be especially attractive for bereaved patients, as they often have to deal with new problems after the loss (e.g., inheritance issues, problems in everyday life as a result of secondary losses). Furthermore, they might be afraid of interventions that foster emotional engagement with the loss because of avoidance or unbearable emotional pain. This might be also the case in patients suffering from PTSD, for whom PCT achieved lower dropout rates than trauma-focused CBT (26, 27). Additional mechanisms underlying PCT may rely on the therapeutic benefits that emerge from a caring relationship, including mobilization of hope and optimism, and increased positive self-regard (67). These are non-specific elements every psychotherapy contains, but they might be activated to a special degree in PCT. However, these assumptions regarding mechanisms underlying PCT and how they affect long-term effects of treatment success require precise evaluation in future process-outcome studies.

### Limitations and Strengths

The generalizability of our study results is limited by the small sample size and the predominantly female (80%) sample. A more general limitation is the lack of a control group, which reduces the strength of the conclusions that can be drawn from the findings. Therefore, natural remission of symptoms or non-specific effects of supportive study attention cannot be excluded. In addition, the non-randomized design may have influenced participants' motivation for taking part in the trial and their retention other than an RCT design. Hence, an RCT with a solid sample size is necessary to test the specific efficacy of PCT for PGD. Furthermore, follow-up assessments for a longer period than 3 months after treatment would have been advisable. Since the ratings of treatment adherence by independent trained raters are not completed yet, we are unable to report on the results of a formalized adherence rating. Yet, specific focus in supervision and case consultations was given to treatment adherence and specifically to the omission of CBT interventions. Finally, using a qualitative methodological approach would have provided further insight regarding therapists' acceptability of PCT. Despite these limitations, which are primarily due to the study design, feasibility trials are of high interest, especially to foster research when there is a lack of controlled studies as for PGD (14). Feasibility trials allow to examine the safety and acceptability of a new or adapted intervention for a new target group before administering it in larger trials. They are also crucial to examine treatment and training protocols. When developing a psychological treatment, it is important to consider how easily and successfully health professionals new to grief treatment can learn to administer it. If a high amount of training is needed to teach the new treatment, dissemination and implementation might prove difficult. Finally, feasibility trials allow for developing adherence/competence measures and recruitment procedures that are crucial for subsequent RCTs, if the treatment demonstrates clinical impact. To address these goals, the current trial served as an important first step in evaluating PCT for PGD and is in line with sample sizes of other pilot and feasibility trials with bereaved patients [e.g., (68–70)].

### Implications

In conclusion, our study furnishes preliminary evidence of the feasibility, efficacy, and safety of PCT with PGD. PCT adapted for PGD was deemed to be an adaptable and easy-to-learn intervention of good design quality by our relatively young CBT therapists. Future studies are needed to address the question of the efficacy of PCT adapted to PGD compared to adequate control conditions. Based on our results, it seems reasonable to choose and evaluate PCT as an active control condition compared to a grief-focused CBT, including exposure and cognitive structuring, in a current RCT (35). Furthermore, the need for specific treatment components, such as exposure, cognitive restructuring techniques, or behavioral activation, should be addressed in dismantling studies targeting patients suffering from PGD. PCT might be a viable alternative for patients unable or unwilling to participate in grief-focused treatments. Further evaluation of PCT in RCTs may help determine which treatment components are beneficial and necessary for the individual PGD patient and might allow formulation of individualized treatment recommendations and different treatment selection [see (71)]. Therefore, further research regarding the efficacy of PCT but also regarding individual predictors of treatment success in PCT is of high interest.

## Data Availability Statement

The datasets generated for this study are available on request to the corresponding author.

## Ethics Statement

The studies involving human participants were reviewed and approved by Institutional Review Board of the Catholic University Eichstaett-Ingolstadt, Ethics Committee of the Department of Psychology and Sports of the Goethe University Frankfurt, Ethical Committee at the Medical Faculty of Leipzig University, Local Ethics Committee of the Department of Psychology of the University of Marburg. The patients/participants provided their written informed consent to participate in this study.

## Author Contributions

AV and RR contributed to the design of the study. RR was the principal investigator. AK, WR, and RS were leaders of the collaborating study centers. HC organized the database. AV performed the statistical analyses and wrote the first draft of the manuscript. AN and RR wrote sections of the manuscript. All authors contributed to the manuscript revision, read and approved the submitted version.

## Conflict of Interest

AN was paid fees for supervising PCT. RR was paid fees for workshops and presentations on PGD treatment. The remaining authors declare that the research was conducted in the absence of any commercial or financial relationships that could be construed as a potential conflict of interest.
