# Supplementary material for: Feasibility of Present-Centered Therapy for Prolonged Grief Disorder: Results of a Pilot Study
Source: Front Psychiatry. 2021 Apr 15;12:534664. doi: 10.3389/fpsyt.2021.534664 (PMC8081969; doi:10.3389/fpsyt.2021.534664)
Supplement: Supplementary file 1 [file Data_Sheet_1.DOCX]

| **Evaluation of Present-Centered Therapy (PCT) for Prolonged Grief Disorder** | | | | | |
| --- | --- | --- | --- | --- | --- |
| Please indicate on a 5-point scale the degree to which you agree with the following statements about PCT (1 = “I do not agree at all“, 5 = “I agree fully“).  Thank you very much! | | | | | |
| **Beliefs and attitudes about the intervention** | | | | | |
| 1. PCT conveys a plausible etiology model of prolonged grief disorder (PGD). | 1 | 2 | 3 | 4 | 5 |
| 1. PCT may be effective in reducing PGD symptoms. | 1 | 2 | 3 | 4 | 5 |
| 1. PCT may be effective in reducing comorbid symptoms, such as somatoform complaints or depressive symptoms. | 1 | 2 | 3 | 4 | 5 |
| 1. PCT increases the patients’ emotional wellbeing. | 1 | 2 | 3 | 4 | 5 |
| 1. With the help of PCT, patients are able to live a satisfactory life without the deceased person. | 1 | 2 | 3 | 4 | 5 |
| 1. With the help of PCT, patients learn skills that enable them to deal appropriately with future difficulties. | 1 | 2 | 3 | 4 | 5 |
| 1. PCT leads to improvements in my patients that I can actually see. | 1 | 2 | 3 | 4 | 5 |
| 1. Using PCT includes a risk of doing more harm than good. | 1 | 2 | 3 | 4 | 5 |
| 1. Using PCT fits well with the way I like to work. | 1 | 2 | 3 | 4 | 5 |
| **Design quality** | | | | | |
| 1. It is quite possible to work through the specified contents in the time specified by the manual. | 1 | 2 | 3 | 4 | 5 |
| 1. PCT is clear and understandable. | 1 | 2 | 3 | 4 | 5 |
| 1. Goals and priorities when administering PCT are clear and consistent. | 1 | 2 | 3 | 4 | 5 |
| **Adaptability and trialability** | | | | | |
| 1. It is easy to try out PCT and see how it performs. | 1 | 2 | 3 | 4 | 5 |
| 1. PCT is easy to use. | 1 | 2 | 3 | 4 | 5 |
| 1. The skills required to implement PCT can be effectively taught. | 1 | 2 | 3 | 4 | 5 |
| 1. PCT can be adapted to meet the needs of my patients. | 1 | 2 | 3 | 4 | 5 |
| **Resources and access to knowledge** | | | | | |
| 1. The PCT manual is helpful. | 1 | 2 | 3 | 4 | 5 |
| 1. PCT has helpful supportive materials for patients. | 1 | 2 | 3 | 4 | 5 |
| 1. There is adequate supervision to support me in implementing PCT. | 1 | 2 | 3 | 4 | 5 |
| 1. There are adequate communication systems (e.g. Dropbox, supervision) to support information exchange with regard to PCT. | 1 | 2 | 3 | 4 | 5 |
| 1. There are barriers to implementing PCT. | 1 | 2 | 3 | 4 | 5 |
| If ≥ 2, please briefly describe the barrier(s): _____________________________________  ________________________________________________________________________  ________________________________________________________________________  ________________________________________________________________________  ________________________________________________________________________  ________________________________________________________________________ | | | | | |
